# An index of Chinese surname distribution and its implications for population dynamics

**DOI:** 10.1002/ajpa.23863

**Published:** 2019-05-29

**Authors:** Jiawei Chen, Liujun Chen, Yan Liu, Xiaomeng Li, Yida Yuan, Yougui Wang

**Affiliations:** ^1^ School of Systems Science Beijing Normal University Beijing 100875 China; ^2^ Institute of Genetics and Developmental Biology Chinese Academy of Sciences Beijing 100101 China

**Keywords:** isonomy, migration, population dynamics, surname distance, surname distribution

## Abstract

**Objective:**

We propose an index to characterize the key feature of Chinese surname distributions and investigate its implications for population structure and dynamics.

**Materials and methods:**

The surname dataset was obtained from the National Citizen Identity Information Center, which contains 1.28 billion Chinese citizens enrolled in 2007, excluding those of Hong Kong, Macao, and Taiwan. An index, the coverage ratio of stretched exponential distribution (CRSED), is proposed based on the crossover point of stretched exponential truncated power‐law distribution, where the stretched exponential term and the power‐law term contribute equally. We use multidimensional scaling technique to demonstrate the dependence of the similarity of one prefecture to the others on the CRSED.

**Results:**

The CRSEDs of 362 prefectures exhibit an uneven distribution. The consistency of this index is evident by strong positive correlations of CRSEDs at the three administrative levels. This new index has a strong negative correlation with the proportion of the rare surnames. The prefectures with similar CRSEDs tend to adjoin each other on the administrative map, resulting in several distinct regions, each of which shares similar terrain features or historical migrations. The prefectures with lower CRSEDs are more dissimilar to the other prefectures, while the ones with higher CRSEDs are more similar to the others.

**Discussion:**

The population dynamics of the prefectures with higher CRSEDs are more likely dominated by migratory movements, the dominant evolutionary forces of the prefectures with lower CRSEDs can be attributed to drift and mutation.

## 1 INTRODUCTION

Surnames are transmitted from father to children in patrilineal society, so they can be taken as an alternative for alleles on the Y‐chromosome. As a kind of characterization of population structure, surname distributions have been extensively investigated by anthropologists, geneticists, physicists, and scientists in many other fields (Darlu et al., 2012; Piazza, Rendine, Zei, Moroni, & Cavalli‐Sforza, 1987; Rossi, 2013; Zei, Guglielmino, Siri, Moroni, & Cavalli‐Sforza, 1983; Zei, Matessi, Siri, Moroni, & Cavalli‐Sforza, 1983).

In most countries and regions, surname distributions are found to follow power‐law in their representation of frequency distribution, cumulative distribution or Zipf plot (Baek, Kiet, & Kim, 2007; Miyazima, Lee, Nagamine, & Miyajima, 2000; Zanette & Manrubia, 2001). However, some different kinds of surname distributions have also been observed. For example, the logarithmic form of the cumulative surname distribution of Korea has remained unchanged for five centuries (Kim & Park, 2005). Similarly, the top 100 most popular surnames in China exhibit an exponential Zipf plot, which has been maintained since the Song dynasty (Baek et al., 2007; Yuan & Zhang, 2002). Nevertheless, the cumulative distributions of surnames on all three levels of province, prefecture, and county in China were found to follow a unified form of stretched exponential truncated power‐law (Chen, Chen, Liu, Wang, & Wang, 2011).

Surname distribution, as an integrative result from the evolutionary forces such as drift, mutation, and migration, contains important information of population dynamics. For example, Pavesi et al. studied the surname distribution of 312 communes in Sicily, where all the distributions could be regarded as power‐law type. However, the fitted exponents varying from 0.46 to 1.83 appeared to be associated with the level of isolation and thus indicating that the relative strength of migratory movements in these communes may govern the population dynamics (Pavesi, Pizzetti, Siri, Lucchetti, & Conterio, 2003). From this result, a question is raised about whether any other key features of surname distribution can also be regarded as an indicator of population dynamics. In this article, we will employ surname distributions in China to address this question.

Chinese surnames are quite suitable for investigating the implications of surname distribution for population dynamics (Chen et al., 2011; Liu, Chen, Yuan, & Chen, 2012; Shi et al., 2018; Shi et al., 2019). Chinese surnames have been well preserved through generations due to the prevalence of Confucian culture, in which people do not change their surnames unless they have to do so (Du & Yuan, 1995; Du, Yuan, Hwang, Mountain, & Cavalli‐Sforza, 1992). This has allowed long‐term random drift to take its function for more than 4,000 years. During the process of random drift, there were also many large‐scale migratory movements in the history of China. As a result, Chinese surnames have experienced long‐term integration between locals and migrants. However, the scale of these immigrations and their effects on the local population are quite different from region to region. And such variations will be definitely embodied in surname distributions. In fact, the cumulative distribution of Chinese surnames follows a unified form of stretched exponential truncated power‐law, but the fitted parameters vary greatly in different regions (Chen et al., 2011), which must be associated with different migratory movements.

In this article, a new index of surname diversity, the coverage ratio of stretched exponential distribution (CRSED), is put forward to characterize the relative importance of stretched exponential term to power‐law term in this kind of surname distribution in Subsection 2.3. That is, a surname distribution with a higher CRSED corresponds to a more stretched‐exponential‐like distribution, while that with a lower CRSED corresponds to a more power‐law‐like form. The implications of CRSED for population structure are thoroughly investigated at the level of prefecture in Section 3.1. Then, three aspects of CRSED are investigated, including the consistency of CRSED at the three administrative levels in Section 3.2, the spatial distribution of CRSED and the corresponding features of geographic environment and historical migratory movements in Section 3.3, and the relevance of CRSED for each prefecture to its degree of surname similarity with other prefectures in Section 3.4. Based on the results, a hypothesis on the relationship between CRSED and population dynamics is put forward and qualitatively explained in Section 4.

## 2 MATERIALS AND METHODS

### 2.1 Data and materials

The surname dataset in this article was obtained from China's identity information system, which was constructed by the National Citizen Identity Information Center. The data contain 1.28 billion people who were enrolled in 2007 and who live in mainland China, excluding Hong Kong, Macao, and Taiwan.

In Chinese naming system, most surnames appear generally the first Chinese character followed by the given name, so the first Chinese character of one's name is taken as his/her surname. However, a few surnames, such as Ouyang (欧阳), Zhuge (诸葛), and Linghu (令狐), consist of multiple characters. In these cases, taking the first Chinese character as surname may result in some inaccuracy. However, this inaccuracy is so slight that it could be dismissed due to the rarity of such kinds of surnames. Further preprocessing, including removing non‐Chinese character surnames and merging the surnames expressed in traditional characters into the corresponding simplified ones, is necessary. After these operations, we obtain a total of 7,184 surnames, which are used in the following analysis.

China is an integrated country of multiple ethnic groups, with the Han as the largest one accounting for 91.4% of the total population and with 55 ethnic minority groups. The naming systems of most ethnic minorities are the same as that of the Han. However, some ethnic minorities have different naming systems or even have no surname at all (Qian, 1989). In the latter case, surnames have been assigned using the first character of their names so that all the surnames can be treated in a consistent way. As a result, surname distributions in the prefectures with a high proportion of these ethnic minorities may be extraordinary.

### 2.2 Previous index on surname structure

Isonomy is one of the most commonly used index in the surname researches. The isonomy within a region *i* is defined as $I_{i}=\sum_{k=1}^{S}p_{\mathrm{ki}}^{2}$, where *p*_*ki*_ is the proportion of the population with surname *k* to the entire population, and *S* is the total number of surnames. The isonomy between two regions *i* and *j* is defined as $I_{\mathrm{ij}}=\sum_{k=1}^{S}p_{\mathrm{ki}}p_{\mathrm{kj}}$. The isonomy within a region characterizes the aspect of within‐population structure, while the isonomy between two regions reveals another aspect of population structure, the between‐population similarity.

The difference in population structure between any two regions can be measured by surname distance. There are several definitions of surname distance, such as Lasker's distance (Rodriguez‐Larralde et al., 1998), Euclidean distance, and Nei's distance (Cavalli‐Sforza & Edwards, 1967). Nei's distance, which can also be taken as a specially normalized form of the isonomy between two regions, is commonly used in relevant works and will be adopted in this article. Specifically, Nei's distance between regions *i* and *j* is defined as $N_{\mathrm{ij}}=-\log\;\left(I_{\mathrm{ij}}/\sqrt{I_{i}I_{j}}\right)$ (Nei, 1972).

The isonomy analysis is helpful for measuring the structure and regional consanguinity of the Chinese population, as shown in previous studies (Du et al., 1992; Yuan, Jin, & Zhang, 1999; Yuan, Zhang, Ma, & Yang, 2000). However, the definition of isonomy implies that the popular surnames have absolute dominance over the less popular ones, so the information contained in the less popular surnames cannot be adequately revealed by isonomy analysis. It is inappropriate above all in the case of China since the 7,000 less popular surnames only account for 6.7% of the population of total 7,184 surnames.

There are several sources of the less popular surnames in a given region. They may be the surnames of local minorities which have maintained at a small size of population for a long time. Maybe they are the relatively new surnames which either mutated recently from the existing surnames of local residents or brought about by foreigners who immigrated from other regions not long ago. Thus, the information contained in the less popular surnames can be especially important for the researches on population structure and population dynamics.

Some other indexes are necessarily required to complement the isonomy analysis in this sense. The ratio of surname to population *S*/*N* can be taken as one of these indexes. Suppose that 10 people with a new surname migrate into Beijing with a population of 11.89 million and 1941 surnames. The migration will have no perceptible impact on the index of isonomy in Beijing since these immigrants account for only one millionth of the population, but it will increase *S*/*N* by about 0.5‰. Specifically, the less popular surnames possibly increase *S*/*N*, while the popular surnames have the opposite impacts. Besides *S*/*N*, the proportion of Hapax is another one of these index. Here, Hapax means the surname with only one person in a region, so it focuses on the least popular surnames instead of the most popular ones.

### 2.3 A new index of surname distribution: CRSED

The cumulative distribution function (CDF) of Chinese surnames for all the administrative levels can be fitted with a stretched exponential truncated power‐law function (Chen et al., 2011), that is,

(1) $$P\left(n\right)=a\cdot n^{-b}\cdot e^{-{\left(n/c\right)}^{d}},$$

where *P*(*n*) represents the proportion of surnames whose sizes are no less than *n*, *b* is the power exponent, *c* is the cutoff size of power‐law part, and *d* is the stretch parameter in the stretched exponential function (Bonabeau, Dagorn, & Fréon, 1999).

Actually, the function exhibits a crossover from the power‐law form to the stretched exponential one. Specifically, the function looks like power‐law in the domain where the value of *n* is small enough, while it will transform into a stretched exponential function when *n* is large enough. Although the parameter *c* is commonly taken as the cutoff size of power‐law, a more justified crossover point will be defined as follows.

The right side of Equation (1) is the product of two terms, the power‐law *n*^−*b*^ and the stretched exponential $e^{-{\left(n/c\right)}^{d}}$, thus the first derivative of the function contains two parts: the one from the first derivative of *n*^−*b*^ and the one from that of $e^{-{\left(n/c\right)}^{d}}$. The relative importance of the power‐law term and the stretched exponential term in this function can be determined by the relative magnitude of their counterparts in the first derivative. According to

(2) $$\frac{dP}{dn}=a\cdot n^{-b-1}\cdot e^{-{\left(n/c\right)}^{d}}\cdot \left[-b-d{\left(\frac{n}{c}\right)}^{d}\right],$$

the crossover point can be reasonably defined as the point where the two derivative parts are equal to each other, thus we can get the expression of the crossover point as follows:

(3) $$n_{0}=c\cdot {\left(\frac{b}{d}\right)}^{1/d}.$$

Specifically, the power‐law form dominates in the domain of *n* < *n*_0_, while the stretched exponential form dominates in the domain of *n* > *n*_0_ (Chen et al., 2011). Combining the definition of *n*_0_ in Equation (3) and the definition of *P*(*n*) as the proportion of surnames whose sizes are no less than *n*, the value of *P*(*n*_0_) represents the proportion of surnames that fall into the domain of stretched exponential form. Thus, *P*(*n*_0_) means that CRSED can be used as an index to characterize the key feature of the stretched exponential truncated power‐law distribution. Generally speaking, a higher CRSED corresponds to a more stretched‐exponential‐like distribution, while a lower CRSED corresponds to a more power‐law‐like one.

In order to estimate the CRSED of a surname distribution, the CDF profile should be used instead of the fitted curve. Specifically, the value of *n*_0_ on the fitted curve will be estimated with the fitted parameters *b*, *c* and *d* according to Equation (3). Then the crossover point has to be set as the ceiling of *n*_0_ and the CRSED on the actual CDF will be the proportion of surnames whose sizes are no less than this crossover point. In the extreme case, if the estimated value of *n*_0_ on the fitted curve is less than one, it has to be set as one and then the CRSED will be set to be 100%. For simplicity, the same symbol *n*_0_ is used to represent the fitted value and its ceiling, and the same index CRSED is used to represent *P*(*n*_0_) of the fitted curve and the real data.

**Figure 1:**
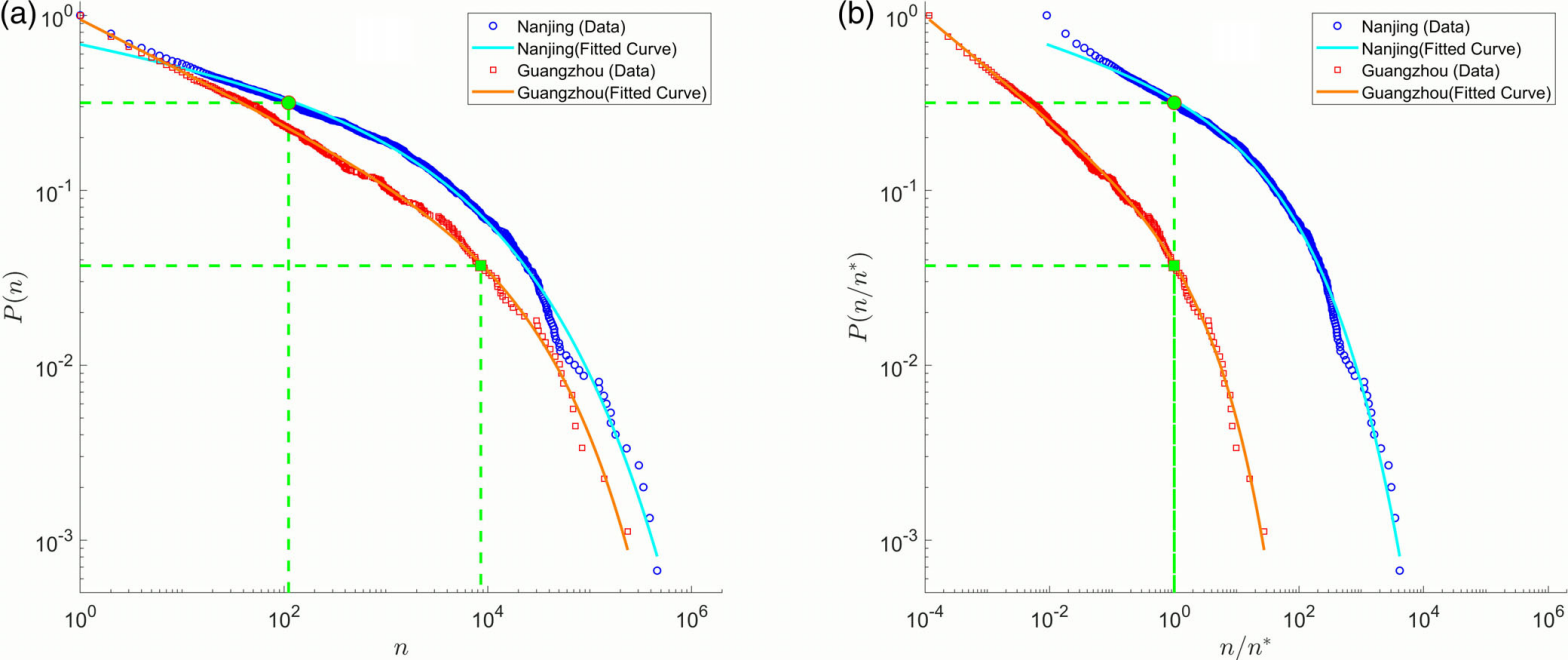
The surname distribution of two typical prefectures. (a) The cumulative distribution functions (CDFs) of surnames of Nanjing and Guangzhou. (b) Normalized CDF of Nanjing and Guangzhou with *n* divided by *n*_0_, respectively

The intuitive meaning of CRSED can be illustrated by those of two typical prefectures, Nanjing and Guangzhou, as shown in Figure 1a. The line of the crossover point *n* = *n*_0_ segments the curve of surname distribution into two sections. The left section represents the domain of less popular surnames which takes a power‐law‐like form, and the right section refers to the domain of relatively popular surnames, which looks like a stretched exponential distribution. The corresponding CRSED represents the proportion of surnames in the right section. The quite large value of CRSED for Nanjing means that a large section of its surname distribution takes a stretched exponential form. Comparatively, the quite small value of CRSED for Guangzhou means that a major section of its surname distribution is dominated by a power‐law‐like form. The difference between these two surname distributions can be illustrated more clearly by the normalized CDF with *n* divided by *n*_0_, as shown in Figure 1b.

## 3 RESULTS

### 3.1 Implications of CRSED for population structure

The CRSED of the whole country is 0.040 with the crossover point of *n*_0_ = 273, 807. Combining a total of 7,184 surnames in the whole country, it represents about 288 relatively popular surnames fall into the domain of stretched exponential form. Next, we will investigate the CRSEDs of 362 prefectures (or prefecture‐level cities, autonomous prefectures, and leagues), which is the level we focus on in this work.

**Figure 2:**
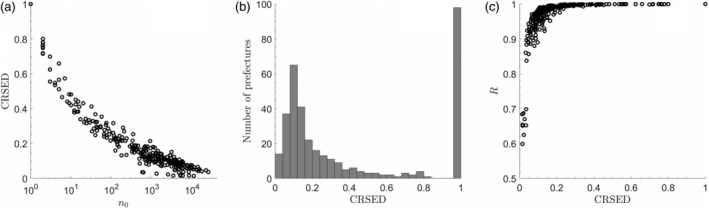
The CRSEDs for the 362 prefectures. (a) The scatter plot of *n*_0_ versus CRSED. (b) The histogram of the CRSEDs. (c) The scatter plot of CRSED versus *R*, which represents the proportion of all the people whose surnames fall into the domain of stretched exponential form to the entire population. CRSED, coverage ratio of stretched exponential distribution

The crossover points *n*_0_ and the corresponding CRSEDs for the 362 prefectures are obtained with the fitted parameters of Equations (1) and (3), respectively. The correspondence between *n*_0_ and the CRSED is shown in Figure 2a and the histogram of the CRSEDs is shown in Figure 2b. The CRSEDs are quite uneven with two peaks around 0.1 and 1. Note that there are 98 prefectures whose CRSEDs are 1. For these prefectures, all the surnames fall into the domain of stretched exponential form. There are 179 prefectures whose CRSEDs are concentrated within the interval of [0, 0.2], and the corresponding values of *n*_0_ are mostly on the order of thousands, ranging from 100 to 10,000. The other 85 prefectures whose CRSEDs approximately decreasingly distribute in the range of [0.2, 0.8], with quite small *n*_0_ on the order of 10, ranging from 2 to 100.

To obtain a comprehensive understanding of the implications of CRSEDs for population structure, it is especially necessary to know the proportion of the people whose surnames fall into the domain of stretched exponential form. Let *R* represent the proportion and the correspondence between CRSED and *R* is shown in Figure 2c. Obviously, the higher the CRSED is, the larger the corresponding *R* is. Specifically, at the one end where the CRSEDs are 1, the corresponding *R* values are 1. At the other end where the CRSEDs are as low as only about 0.1, the corresponding *R* values are around 0.95. The comparison of these two ends indicates that although the differences in *R* among the prefectures are as small as about 0.5, the differences in CRSEDs can be as prominent as 0.9. In other words, the CRSED, as the proportion of surnames fall into the domain of stretched exponential form, can be insensitive to the corresponding population proportion. Thus, the CRSED can be used to reveal the information contained in the less popular surnames that are quite essential in population dynamics.

**Figure 3:**
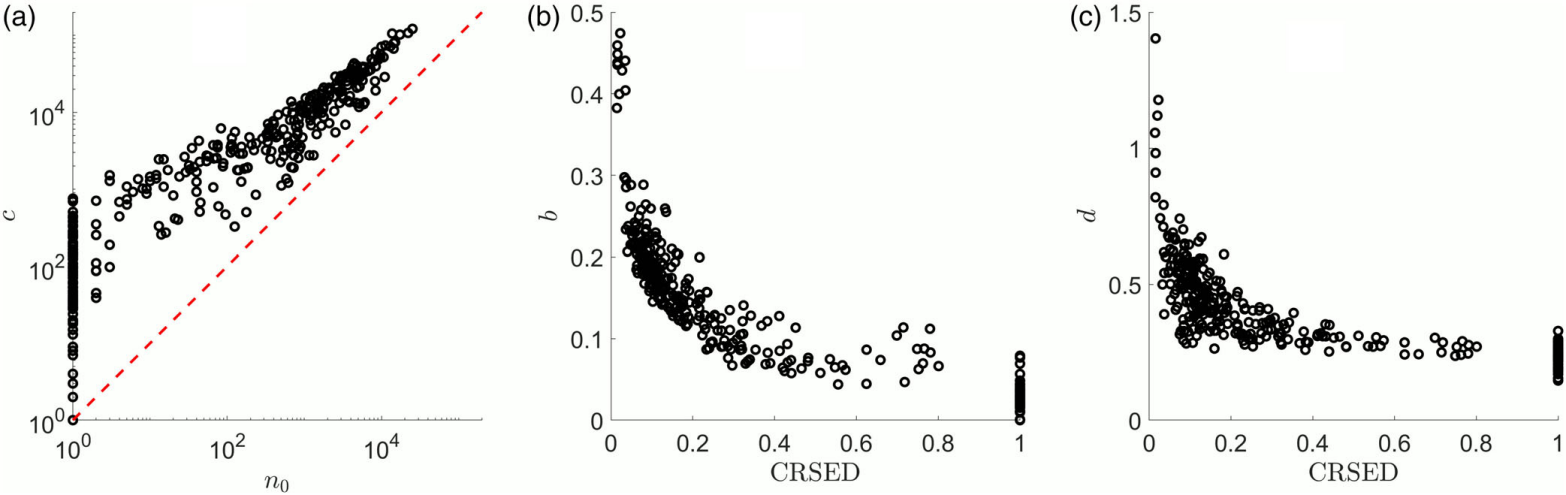
The fitted parameters for the 362 prefectures. (a) The correlation between *n*_0_ and cutoff size *c*. The dashed line represents that *n*_0_ = *c*. (b) The correlation between CRSED and power exponent *b*. (c) The correlation between CRSED and stretch exponent *d*. CRSED, coverage ratio of stretched exponential distribution

The correlations between the fitted parameters in Equation (1) and the CRSED or *n*_0_ are also important to understand the new index, especially the necessity of introducing *n*_0_. According to Equation (3), *n*_0_ is determined by the three fitted parameters such as *b*, *c*, and *d*. There is a significantly positive correlation between *n*_0_ and the cutoff size *c*, while the former is roughly one order of magnitude smaller than the latter as shown in Figure 3a. However, *c* varies greatly at the part of small *n*_0_, such as *n*_0_ < 100, where most of the corresponding CRSEDs are more than 0.2 as shown in Figure 2a. In this range of CRSED, the corresponding power exponents *b* are <0.1 and the stretched exponents are relatively stable from 0.3 to 0.4 as shown in Figure 3b,c. That is, in the CDFs for these prefectures, the power‐law part has been overwhelmingly dominated by the stretched exponential part. However, such inference cannot be directly inferred by the fitted parameter *c*. Take the prefectures with *n*_0_ = 1_,_ for example, the CDFs for these prefectures can be well fitted by a stretched exponential function according to the CRSEDs, but such inference cannot be obtained if the crossover points are only characterized by the fitted parameters *c* as they range from 1 to 100.

**Figure 4:**
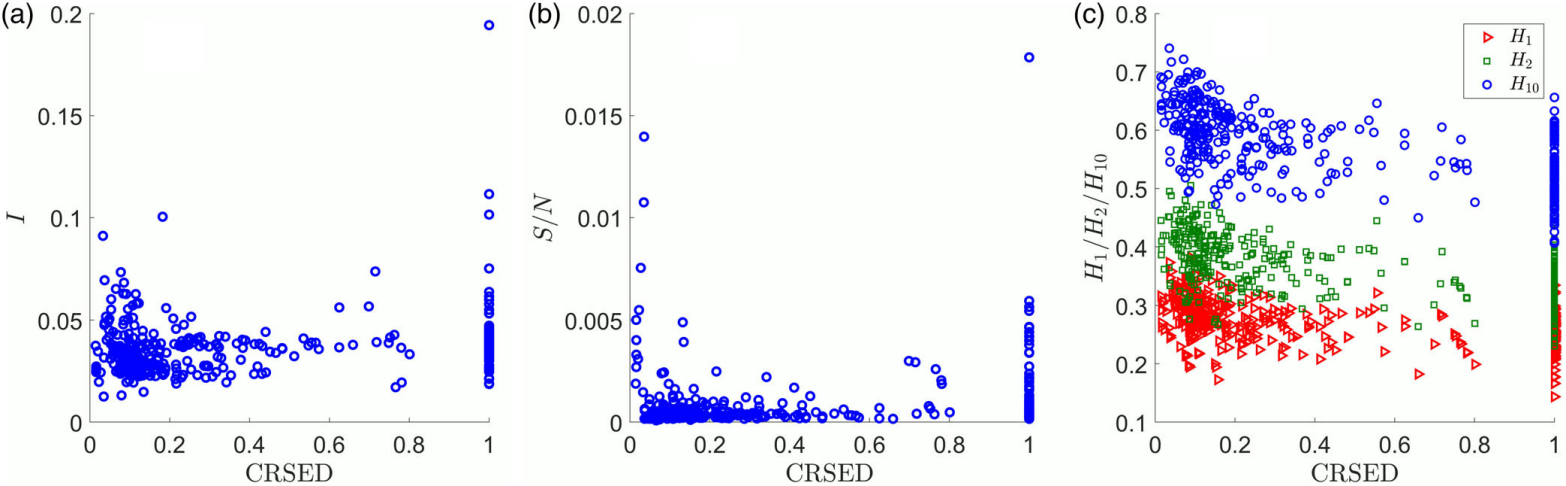
The correlation between CRSED and some other indexes. (a) The scatter plot of CRSED versus *I*. (b) The scatter plot of CRSED versus *S*/*N*. (c) The scatter plot of CRSED versus *H*_1_, *H*_2_, and *H*_10_. The red triangles represent the high rarity level of surname *H*_1_, the green squares represent the median rarity level *H*_2_, and the blue circles represent the low rarity level *H*_10_. CRSED, coverage ratio of stretched exponential distribution

The implications of CRSED for population structure can be further revealed by the comparison of CRSED with isonomy *I*, an index commonly used in surname analysis. The scatter plot of CRSED versus *I* for 362 prefectures is shown in Figure 4a. There is a weakly positive correlation between the two indexes, and the Spearman correlation coefficient is .20. Thus, this new index CRSED is quite different from isonomy and can infer something that cannot be revealed by isonomy. It is reasonable as isonomy is mainly determined by the popular surnames while CRSED is a key feature of the surname cumulative distribution.

As pointed out above, the CRSED can be used to reveal information contained in the less popular surnames. Here, the correlation between CRSED and the index related to the less popular surnames should be investigated. Let us consider the ratio of surname to population *S*/*N*. As shown in Figure 4b, there is a weakly positive correlation between CRSED and *S*/*N*, where the Spearman correlation coefficient is .25. Therefore, CRSED can be used to reveal something quite different from the ratio of surname to population. Then let us consider three indexes related to Hapax, the proportions of the rare surnames at three rarity levels. Let *H*_1_, *H*_2_, and *H*_10_ represent the proportion of the surnames with only one person (Hapax), with no more than 2 people, and with no more than 10 people, respectively. The correlation between CRSED and *H*_1_, *H*_2_, and *H*_10_ are shown in Figure 4c, respectively. There are relatively strong negative correlations and the Spearman correlation coefficients are −.50, −.54, and −.60 for *H*_1_, *H*_2_, and *H*_10_, respectively. Generally, the prefectures with higher CRSED have lower proportions of the rare surnames. Thus, CRSED can be used to reveal some useful information in the rare surnames. It is quite interesting but a more detailed discussion is beyond the scope of this article.

### 3.2 Consistency of CRSED

An indirect test for the validity of CRSED in distinguishing the surname distributions is carried out by checking the consistency of CRSED among the three administrative levels. Province is the highest level and each province consists of one or more prefectures. Four special administrative divisions, Beijing, Tianjin, Shanghai, and Chongqing are treated as both province and prefecture. County is the lowest administrative level and several counties constitute a prefecture. Thus, besides the surname distributions at the prefecture level mentioned above, the distributions at the other two levels will be also investigated.

**Figure 5:**
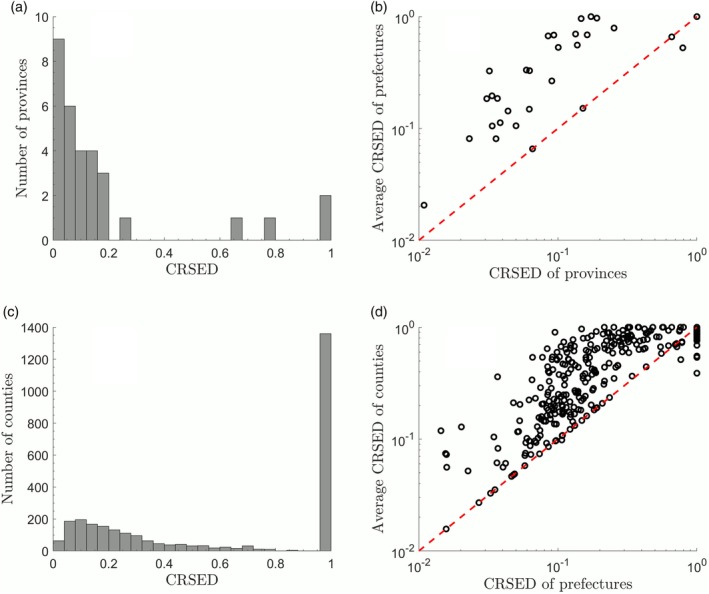
The CRSEDs at the province level and the county level. (a) The CRSED histogram of 31 provinces. (b) The logarithmic scatter plot of the CRSED of a province versus the average CRSED of prefectures within the corresponding province. The four points on the diagonal line are the special administrate divisions. (c) the CRSED histogram of 2,832 counties. (d) The logarithmic scatter plot of the CRSED of a prefecture versus the average CRSED of counties within the corresponding prefecture. CRSED, coverage ratio of stretched exponential distribution

The histogram of CRSEDs for all the 31 provinces (or municipalities, autonomous regions, or special administrative regions) is shown in Figure 5a. There are nine provinces whose CRSEDs are <0.04 and only two provinces whose CRSEDs are 1. By comparing the histogram in Figure 5a and that in Figure 2b, it can be inferred that the histogram of CRSED at the province level is more concentrated at the lower part, implying that the surname distributions are more power‐law‐like form compared to those at the prefecture level. In contrast, the case at the county level is opposite as shown in Figure 4c, where the CRSED histogram of the 2,832 counties are more concentrated at the higher part. There are 63 counties whose CRSEDs are <0.04 and 1,360 counties whose CRSEDs are 1. Thus, there is an evident trend that the CRSEDs at higher administrative level are relatively lower, implying that the surname distributions at the higher level are more power‐law‐like form.

In order to check the consistency of CRSED between the province level and the prefecture level, the average CRSED of the prefectures within each province is calculated. There is a positive correlation between the CRSEDs at the province level and the average CRSEDs at the prefecture level as shown in Figure 5b. The Spearman correlation coefficient is .79. Thus, the prefectures within a province with a lower CRSED are more likely to have relatively lower CRSED and vice versa. This indicates a consistency of CRSEDs at the provincial and prefectural levels. Such consistency can be further confirmed by the comparison between the prefecture level and the county level. As shown in Figure 5d, there is also a positive correlation between the CRSEDs at prefecture level and the average CRSEDs of counties within each prefecture and the Spearman correlation coefficient is .88.

Overall, it can be concluded that the CRSEDs are qualitatively consistent at the three administrative levels and thus the CRSED can be regarded as a valid index in characterizing surname distributions in China.

### 3.3 Geographical representation of CRSED

**Figure 6:**
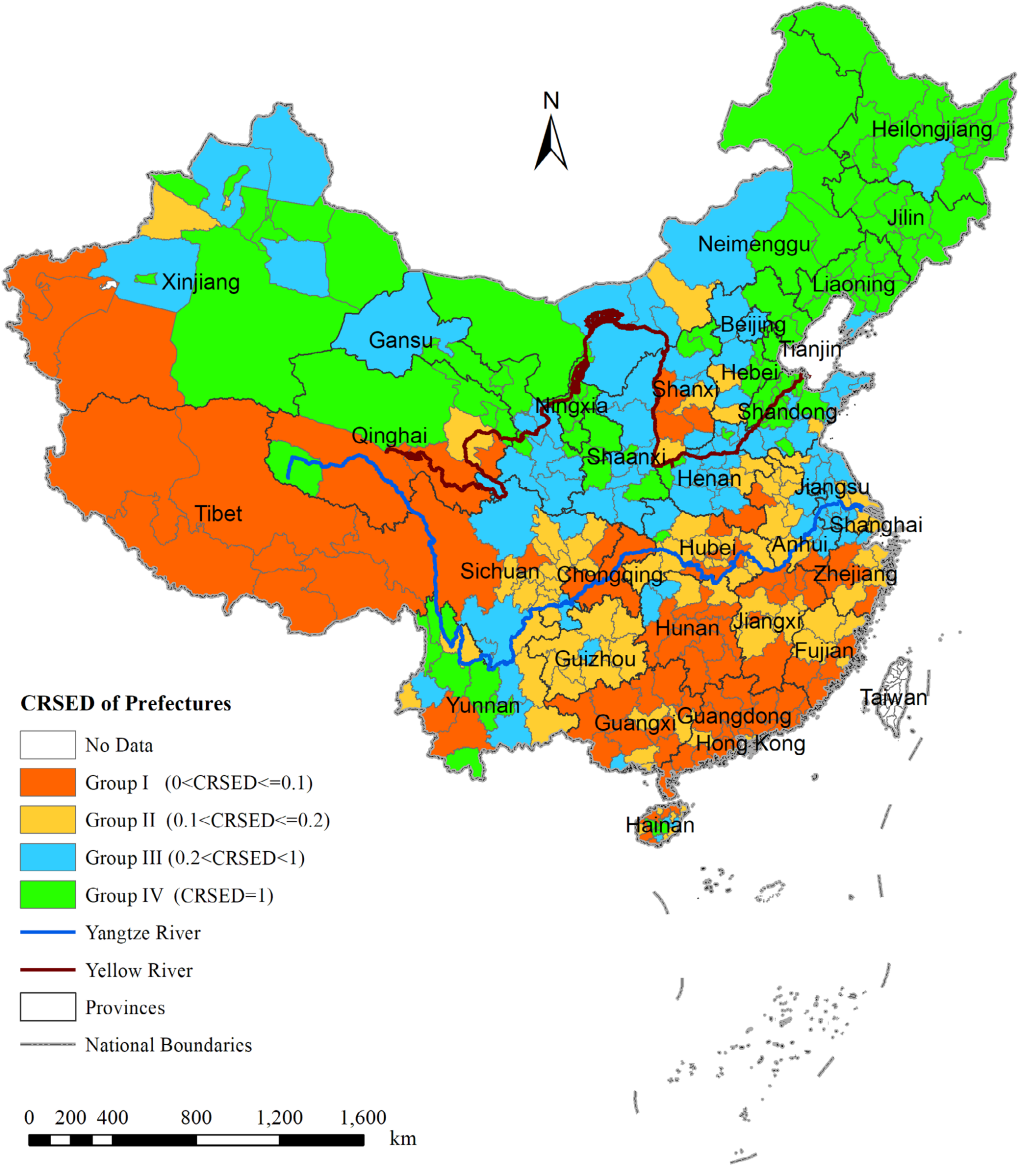
The geographic representation of the CRSEDs for 362 prefectures. CRSED, coverage ratio of stretched exponential distribution

The geographical distribution of the CRSEDs at the prefecture level on Chinese administrative map is represented in Figure 6. For the sake of clear graphical demonstration and meaningful comparison, all the 362 prefectures are classified into four groups according to their CRSEDs and the prefectures in the same group are assigned the same color. The prefectures with the CRSEDs of [0, 0.1], [0.1, 0.2], [0.2,0.9], and 1 are classified as Groups I, II, III, and IV, respectively. Thus, Group I represents the prefectures whose surname distributions look most like a power‐law function, while Group IV represents the ones whose surname distributions are almost stretched‐exponential function.

The spatial association of the CRSEDs can be easily obtained by this way. Strikingly, an explicit pattern appears that the prefectures in the same group tend to adjoin each other geographically. As a result, the prefectures in each of the four groups form a distinct geographical region with only a few outliers. Furthermore, Groups I, II, III, and IV are located in the map from the south to the north sequentially, with the CRSEDs of the corresponding prefectures increasing gradually in this direction.

Next, the general features of each group will be explained, including geographic environment and historical background, especially long‐term migratory movements.

The prefectures in Group I are mainly located in the south and west of China. In most of these prefectures, the terrain is mountainous or hilly, the population contains a relatively high proportion of ethnic minorities, and the local language embodies a specific dialect. Therefore, the people in each of these prefectures are relatively isolated from those living in neighboring prefectures. Due to that, there have been relatively fewer migratory movements between these prefectures and others according to the historical records. Thus, the prefectures in Group I can be taken as the “Isolated Region” hereafter.

The prefectures in Group II are mostly distributed in central and southern China along the middle‐lower reaches of the Yangtze River. The Yangtze River basin is a land abundant in water resources and products, making it very suitable for humans to live in. For long stretches of history, especially after the Song dynasty in the 11th century, people in northern China continued to move from the Yellow River basin to the Yangtze River basin, forming another center of the population there (Tian, 1998). After these long‐term, continuous immigrations, a mixture of populations with different origins had accumulated in the Yangtze River basin, resulting in the highest level of surname diversity (Liu et al., 2012). Therefore, this region can be named the “Immigration Region.”

Most prefectures in Group III are situated in central and northern China along the Yellow River basin. It is well known that the Yellow River basin was the core birthplace of Chinese civilization, as it housed most of the capital cities of ancient empires before the Song dynasty, including Xi'an, Luoyang, and Kaifeng. However, since the Song dynasty, there have been continuous and massive emigrations to other areas, such as “Moving the capital to Lin'an” during the buildup of the Southern Song dynasty. Therefore, this region can be regarded as the “Emigration Region.”

The prefectures in Group IV are mainly located in the northeast and northwest of China. Due to the frigid climate, there was a rather small population in northeast China until the Qing dynasty, and most of the current inhabitants came from the Yellow River basin during the migratory movement of “Braving the journey to the northeast of China” or “Rush to Northeast” in the last two centuries (Fan, 2005). Regarding the northwest of China, although the famous Silk Road was there, its population has also remained small due to the desert climate. However, the recent centuries have witnessed rapid population growth due to a series of migratory movements, including the “Dispatchment to the northwest of China” from the Yellow River basin as well as from other places in the 1950s and 1960s. Since these migrations were initiated to reclaim wasteland, the region can be called the “Reclaimed Region.”

### 3.4 CRSED and surname distance

All the above analysis on CRSED characterizes the surname structure within a given prefecture. Next, the relevance of CRSED for each prefecture to its degree of surname similarity with other prefectures will be addressed.

The (dis)similarity of surname structure between any two prefectures can be measured by their surname distance. In order to show surname distances among all the prefectures graphically, a nonlinear dimensionality reduction technique, multidimensional scaling (MDS), will be used. MDS technique can place each object in low‐dimensional space and preserve the between‐object distances as well as is possible (Kruskal, 1964). With Nei's surname distance matrix among the prefectures as the input, each prefecture will be represented as an object on a two‐dimensional space by MDS technique so that the prefectures with smaller Nei's distances are more likely to be close to each other.

**Figure 7:**
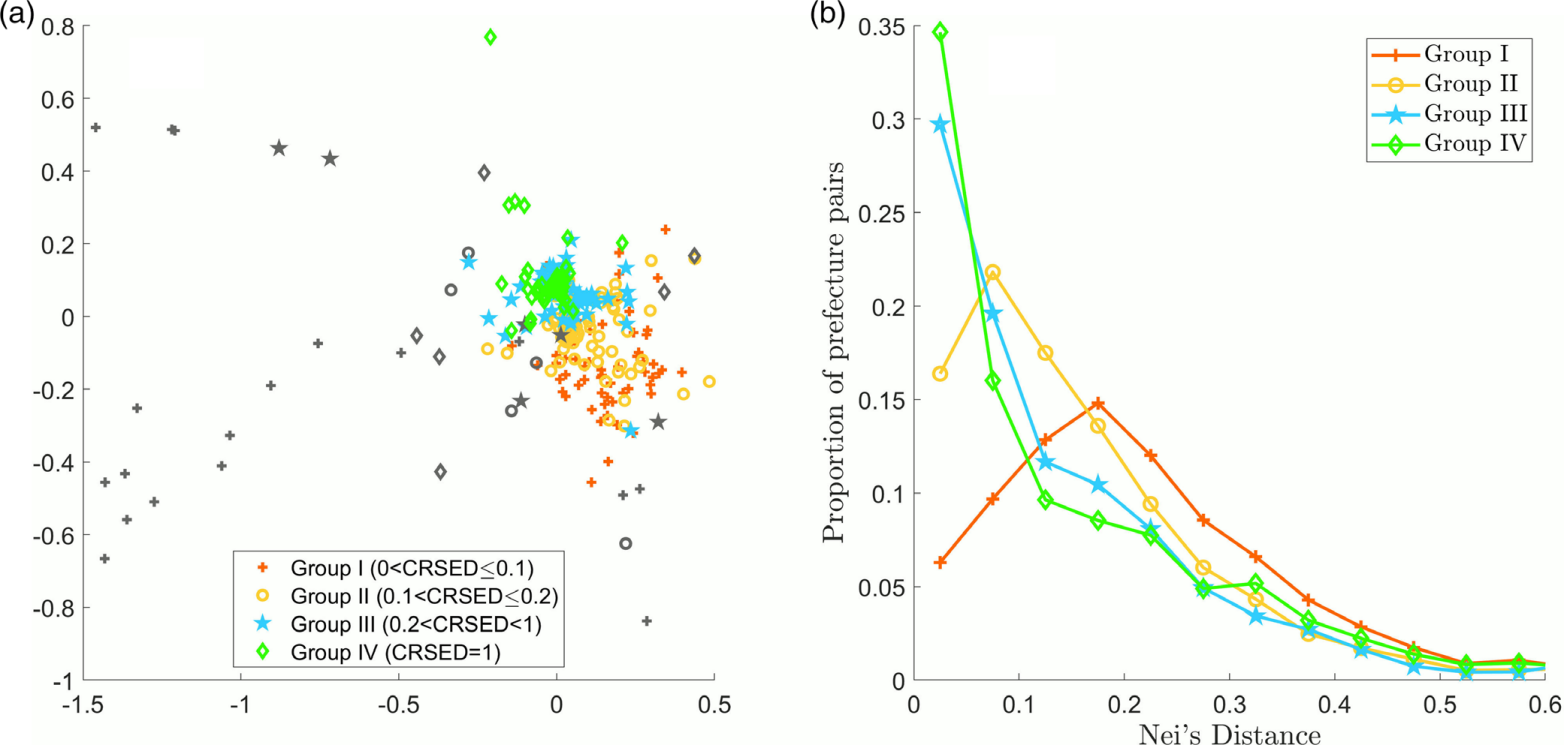
Nei's distance among the 362 prefectures. (a) The 362 prefectures in a two‐dimensional space obtained by multidimensional scaling technique based on Nei's distance. The 35 prefectures that have more than 60% ethnic minorities are marked in gray. (b) The frequency distributions of the pooled Nei's distances for Groups I, II, III, and IV, respectively

There is an evident feature on the two‐dimensional space of Nei's distance as shown in Figure 7a. The prefectures in Groups I and II are scattered around on the graph, while most prefectures in Groups III and IV are relatively clustered together around the center. Moreover, there are some prefectures as extraordinary outliers and most of them are heavily populated by ethnic minorities as shown Figure 7a, where the prefectures with more than 60% ethnic minorities are represented in gray. This isolation from others may result from the inevitable inconsistency between their own naming system and the specific method used to exact the surnames from their names. As the prefectures close to each other have relatively high similarity of surname structure and the outliers are quite different from any of other prefectures, there is a possible correspondence between CRSED and (dis)similarity of surname structure: the prefectures with lower CRSEDs (Groups I and II) are more dissimilar to the other prefectures, while the ones with higher CRSEDs (Groups III and IV) are more similar to the others.

Such correlation can be further justified by the following results. For a given group, the Nei's distances between each prefecture in the group and all the other 361 prefectures are pooled, thus a set of Nei's distances will be obtained for the group. The frequency distribution of the pooled Nei's distances for each group is shown in Figure 7b. The frequency distribution for Group I concentrated at a high value about 0.2, implying that the surname structure of a prefecture in Group I is generally more dissimilar to other prefectures. On the contrary, the pooled Nei's distances for Group III or IV distribute at much lower values, representing that a prefecture in Group III or IV is generally similar to the others.

## 4 DISCUSSION

### 4.1 Implications of CRSED for population dynamics

As mentioned above, the prefectures with higher CRSEDs are more similar to other prefectures, while the ones with lower CRSEDs are more dissimilar to the others. The reason for the similarity between two areas can be frequent or large‐scale migratory movements between them, or that some people in the two areas are immigrated from the same origin. In any of these cases, it can be inferred that if the surname structure in a given area is quite similar to other areas, then migrations probably play a key role in population dynamics in the area. Thus, a hypothesis on the relationship between CRSED and population dynamics is put forward: In the prefectures with higher CRSEDs, migratory movements more likely dominate in population dynamics, whereas in the ones with lower CRSEDs, drift and mutation can be the dominant evolutionary forces.

This hypothesis can be partially supported by a reexamination of the general features of Groups IV and I as mentioned in Section 3.3. Most prefectures with the CRSEDs of 1 in Group IV seem to be remote frontiers and were recently reclaimed by a large number of immigrants from various places, just as called the Reclaimed Region. It can be inferred that multiorigin immigrations have probably played a key role in population dynamics in these areas. A special example is an area in Yunnan province in southwest China as a remarkable outlier in the geographic locations of Group IV. This area is full of high mountains and dense forests, far away from the political center, and is hard to move into. However, approximately 1 million political migrants were organized to consolidate the southwest frontier of China in the Ming dynasty, and many migrants moved into this area for economic reasons in the Qing dynasty. By the end of the 19th century, the Han proportion of the population in Yunnan had almost doubled to nearly 60%, close to the present proportion in this province (Luo, 2013).

In contrast, the prefectures with quite low CRSEDs in Group I seem geographically or culturally isolated, just as called the Isolated Region. It implies that drift and mutation are the dominant forces of population dynamics in these areas. A notable example is an area in Shanxi province in central China is a geographic outlier in Group I. Shanxi province differs from most of the northern plain regions, as it is surrounded by five mountains with several separate basins distributed throughout the area. The relatively closed environment has protected this area from most historical war and disasters, resulting in long‐term stability for the society, a prosperous economy and steady population growth. Although there were recent large‐scale migrations from Shanxi to other parts of China, such as the “Going to the West Gate” (Duan & Gao, 2006), historically, very few people have moved to Shanxi province due to the barrier of mountains.

### 4.2 Qualitative explanation

The relationship between CRSED and population dynamics in China can be qualitatively interpreted by the simple model of population dynamics proposed by Baek et al. (2007), who argued that the difference in surname distributions may originate from the difference in the appearance of new surnames. That is, if the number of new surnames generated per unit of time is proportional to the population size, the power‐law distribution can be derived, whereas if new surnames appear linearly in time irrespective of the total population size, the logarithmic distribution of surnames can be obtained.

More specifically, for prefectures in Group I (or Isolated Regions) whose surname distributions look most like power‐law function, the main source of new surnames should be mutation from local residents. Thus, it is reasonable to presume that the rate of appearance of new surnames is proportional to the population size, resulting in a power‐law‐like surname distribution in these prefectures according to the model. On the contrary, for prefectures in Group IV (or Reclaimed Regions) whose surname distributions are almost stretched‐exponential function, the population consists of a large portion of migrants who could bring new surnames into the area. Since most migratory movements were driven by external forces, the rate of new surnames from migrants should be irrespective of the local population size; thus, the prerequisite for a power‐law surname distribution is violated. Additionally, most immigrations at the prefectural level were discontinuous; thus, new surnames likely appeared nonlinearly in time, disobeying the prerequisite for a logarithmic surname distribution as well. As a result, the surname distributions in these areas must follow a new kind of function. This arouses the question of why the surname distributions in Reclaimed Regions follow stretched‐exponential function. This issue is too complicated to be simply modeled because migrants may not only bring new surnames but also increase the population size of some existing surnames, which are explicitly irrelevant to the local surname composition.

It has to be pointed out that the relationship between CRSED and population dynamics can be only taken as a hypothesis at this stage. It seems true in China, but before it can be regarded as a general rule, much more convincing evidences and theoretical attempts are required in the future.
